# Working conditions and mental health of migrants and refugees in Europe considering cultural origin– a systematic review

**DOI:** 10.1186/s12889-024-18096-7

**Published:** 2024-03-01

**Authors:** Regina Herold, Marietta Lieb, Andrea Borho, Amanda Voss, Susanne Unverzagt, Eva Morawa, Eva Rothermund, Yesim Erim

**Affiliations:** 1 Department of Psychosomatic Medicine and Psychotherapy, University Hospital of Erlangen, Friedrich-Alexander University Erlangen-Nürnberg (FAU), Erlangen, Germany; 2 Institute and Outpatient Clinic of Occupational, Social, and Environmental Medicine, Friedrich-Alexander University Erlangen, Erlangen, Germany; 3 Center of Health Sciences, Institute of General Practice and Family Medicine, Martin-Luther University of Halle-Wittenberg, Halle, Germany; 4 Department of Psychosomatic Medicine and Psychotherapy, Ulm University Medical Center, Ulm, Germany

**Keywords:** Collectivism, Cultural origin, Europe, Individualism, Mental health, Migrants, Refugees, Well-being, Working conditions

## Abstract

**Background:**

Migrants and refugees/asylum seekers, as a large part of the European work force, are often confronted with unfavorable working conditions in the host country. Main aim of this systematic review was to compare the association of these working conditions with mental health between migrants and refugees/asylum seekers due to their diverse migration experiences and cultural origins, and between different European host countries.

**Methods:**

Systematic search for eligible primary studies was conducted in three electronic databases (PubMed/MEDLINE, PsycINFO and CINAHL) using quantitative study designs written in English, German, French, Italian, Polish, Spanish or Turkish and published from January 1, 2016 to October 27, 2022. Primary health outcomes were diagnosed psychiatric and psychological disorders, suicide and suicide attempts, psychiatric and psychological symptoms, and perceived distress. Secondary health outcomes were more general concepts of mental health such as well-being, life satisfaction and quality of life. Two reviewers independently completed screening, data extraction and the methodological quality assessment of primary studies using the Newcastle-Ottawa-Scale. Descriptive summary of primary studies on working conditions and their relationship with mental health were conducted, comparing migrants and refugees/asylum seekers, migrants and refugees/asylum seekers of different cultural backgrounds (collectivistic and individualistic) and migrants and refugees/asylum seekers living in different host countries.

**Results:**

Inclusion criteria were met by 19 primary studies. Voluntary migrants are more likely to experience overqualification in the host country than refugees. In all examined host countries, migrants and refugees suffer from unfavorable working conditions, with migrants from collectivistic countries being slightly at risk compared to migrants from individualistic countries. Most unfavorable working conditions are related to poor mental health, regardless of migrant status, cultural origin or host country.

**Conclusions:**

Although the results should be interpreted with caution due to the small number of studies, it is evident that to maintain both the mental health and labor force of migrants and refugees/asylum seekers, their working conditions in host countries should be controlled and improved. Special attention should be paid to specific subgroups such as migrants from collectivistic societies.

**Ethics and dissemination:**

This systematic review is excluded from ethical approval because it used previously approved published data from primary studies.

**Trial registration number:**

CRD42021244840.

**Supplementary Information:**

The online version contains supplementary material available at 10.1186/s12889-024-18096-7.

## Background

By mid-2020 nearly 281 million persons could be identified as international migrants worldwide [1]. By the end of 2021 of those international migrants, 31.7 million represented refugees and asylum seekers [2]. A person who has changed his or her country of residence is referred to as an “international migrant” [3]. Motives of migration can be, e.g. work, family reunification, higher education (voluntary migration), martial conflicts, persecution or catastrophes (forced migration). Refugees are defined as persons with a forced migration history. Those persons with forced migration experience who have not yet been granted official refugee status are called asylum seekers [4].

While research has already revealed that migrants face disadvantages in working conditions in contrast to natives such as mainly being employed in low-skilled jobs [5, 6], receiving lower payment [6, 7], and facing greater risk of health hazards at work [7–9], to our knowledge no systematic review has focused on the differences in working conditions and their association with mental health between specific migrant subgroups. The different countries of origin of migrants and refugees/asylum seekers in European host countries present a very diverse picture. Migration from one European country to another is described as the largest migration corridor worldwide, while many migrants from other continents also emigrate to Europe [10]. These diverse regions of origin constitute countries of different cultural societies. One of the most widely used classifications of cultures was developed by Hofstede which distinguishes collectivistic from individualistic societies using the Individualism Score. While individualistic societies display loose ties between individuals, life in collectivistic societies takes place in strong, cohesive in-groups [11]. Those cultural backgrounds might affect working life. For instance, individualistic and collectivistic individuals differ regarding work-related needs and conditions in order to achieve the best possible performance. This was shown in an experimental study in which collectivist subjects performed best when they worked anonymously in groups and were weakest when they worked alone and their performance could be traced back to them. In contrast, subjects with an individualistic background showed best performance when their work was traceable. They performed weakest when they worked within a group and their performance could not be traced back to them [12]. Differences in the quality and level of education in non-European countries in contrast to European countries should also be mentioned [13, 14]. Education in a non-European country might lead to disadvantageous working conditions in a European host country. As these differences based on different countries of origin might illustrate the different needs and statuses of migrants and refugees/asylum seekers at work in the host country, cultural backgrounds must be taken into account to prevent the manifestation of mental burdens.

Furthermore, also voluntary migrants and refugees differ in labor market aspects. Compared to natives and other migrant groups refugees are more likely to experience disadvantages in the work context [15]. For instance, refugees were shown to be twice as likely to be unemployed as other migrants [16]. Furthermore refugees/asylum seekers are exposed to other pre-migratory conditions than migrants due to the potentially traumatic flight experience [17] and the subsequent asylum process [18]. Those experiences make them more vulnerable to mental health issues [17–19]. In order to provide effective assistance to maintain or enhance the mental health of migrant and refugee/asylum-seeking workers, it has to be matched to their unique needs. Therefore, potential disadvantages in working conditions of those specific subgroups and their association with mental health have to be identified separately.

Europe is one of the regions with the highest rate of migrants (87 million) [10, 20], since high-income countries have been identified as the main migration destinations [21]. Migrants make up almost 12% of the European population [22] which highlights the (mental) health of migrants in European countries as an important aspect of public health. Among the employed population in Northern, Southern and Western Europe, 18.4% are migrant workers which makes them an important part of the labor force [23]. While a similar direction regarding migration and integration policies of the European Union (EU) member states can be identified [24], they show significant differences to other Western countries such as the so-called classic immigration countries USA, Canada and Australia [25]. But differences between countries within the EU can also be partially found which are due to heterogeneous migration histories. France, Germany, Austria and Belgium, for instance, are considered “traditional migration countries”, whereas Italy, Greece and the Czech Republic have been affected by greater immigration flows only since the 1990s. These differences could influence the labor market integration of migrants and refugees/asylum seekers in the host country [24]. Given these potential differences between European countries, working conditions of migrants and refugees/asylum seekers and their association with mental health should be considered separately for the different European host countries. To the best of our knowledge, no systematic review has yet been conducted in this context with a particular focus on solely Europe. In order to obtain as up-to-date a picture as possible of the work situation and mental health of migrants and refugees in Europe, the focus was placed on the period following the large migration waves to Europe in the years 2014 to 2016 [26].

### Objectives

This systematic review adds evidence to our recent publication on the relationship of working conditions and mental health comparing migrants and refugees/asylum seekers vs. natives [6]. The aim of this systematic review was to compare the relationship between working conditions and mental health (1) between voluntary migrants and forcedly migrated refugees/asylum seekers, (2) between migrants and refugees/asylum seekers from individualistic and collectivistic cultural origins and (3) between different European host countries using a geographic definition [27].

## Materials and methods

This systematic review bases on a published protocol where more information about the specific exclusion and inclusion criteria as well as further methodological procedures can be found (CRD42021244840) [28]. It uses content and structure according to the “Preferred Reporting Items for Systematic Reviews and Meta-Analyses (PRISMA) 2020 statement” [29].

### Systematic search

PubMed/MEDLINE, PsycINFO and CINAHL, were systematically and independently searched. Further, reference lists of included primary studies and relevant reviews were screened. In addition, an unsystematic search on Google Scholar (www.scholar.google.de) was performed. On March 16, 2021, the first literature search for studies published on or after January 1, 2016, was independently conducted by R.H. and F.W. On October 27, 2022, an update was performed by R.H. and A.B. The search strategy included the following three search term clusters: (1) terms related to the study population such as “migrant*” or “refugee*”, (2) terms related to working conditions such as “employ*” or “work*”, and (3) terms related to mental health outcomes such as “mental disorder*” or “well-being” (Supplement 1).

### Study selection and data extraction

The systematic screening of titles, abstracts and full-texts of the primary studies as well as the additional unsystematic search was independently performed by two reviewers (R.H. and F.W./A.B.). Disagreements were discussed between two reviewers (R.H. and F.W./M.L.). In case of disagreement a third/fourth author (Y.E. and E.M.) was consulted.

### Critical appraisal of primary studies

The quality of the included primary studies was independently assessed by the two reviewers (R.H. and F.W./M.L.) using the “Newcastle Ottawa Quality Assessment Scale” (NOS) [30] adapted for cohort studies and cross-sectional studies. Scores between 0 and 9 can be assigned (0–3: “low quality”, 4–6: “moderate quality”, 7–9: “high quality” [31]). Additionally, the two reviewers independently rated the outcome measurement instruments according to whether they were used in the original language in which they were validated, or whether a translation or a culturally adapted version was used.

### Changes to the study protocol

During the conceptualization phase of this systematic review, in addition to the aforementioned comparisons, it was planned to compare the working conditions of migrants and refugees/asylum seekers and their relationship to mental health with those of natives. In order to make the systematic review more concise and thus easier to understand, it was decided to split it into two individual publications. Using the same search strategy, the previously published article exclusively compared working conditions and their association with mental health between migrants/refugees and natives [6], while the present study considered subgroups (migrants vs. refugees, migrants/refugees from individualistic vs. collectivistic countries of origin, different host countries) in terms of the mentioned association.

## Results

### Study selection

**Fig. 1:**
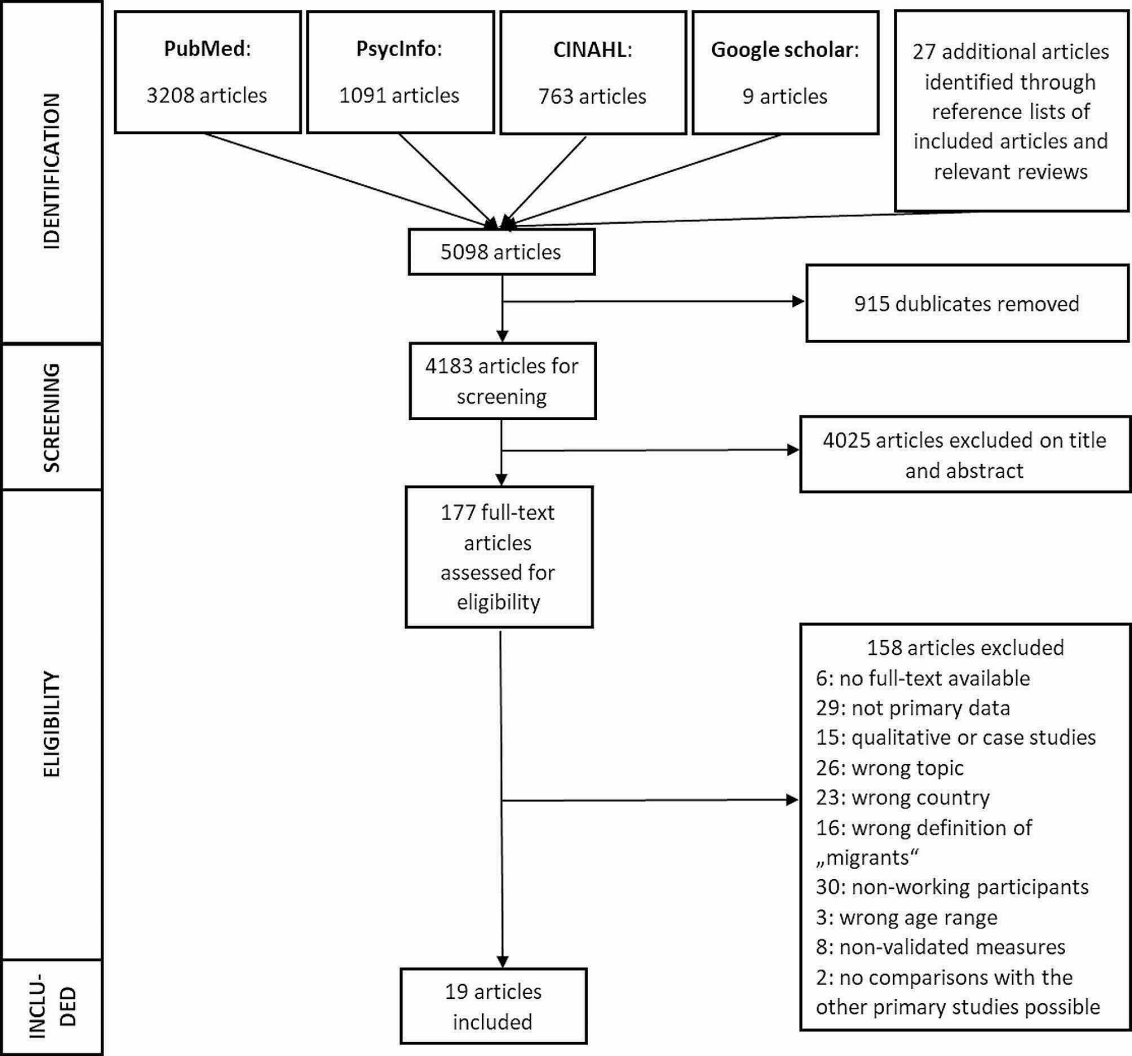
Flow diagram of study selection

The first literature search resulted in 3 722 articles in PubMed (*n* = 2 349), PsycINFO (*n* = 802), and CINAHL (*n* = 571). The second literature search yielded 1 340 articles in PubMed (*n* = 859), PsycINFO (*n* = 289) and CINAHL (*n* = 192). In total, 915 duplicates were removed. Within the scope of the unsystematic search nine additional articles were found, so that 4 183 items were identified for screening. Based on exclusion criteria 4 162 articles were excluded. A total of 177 full-texts were searched and 21 primary studies fulfilled our inclusion criteria and were therefore included in our first systematic review [6]. Two of the primary studies examined the same sample, considering different questions, as well as very special working conditions of natives and migrants of unknown origin [32, 33]. Since they could not be included in the comparisons with the other primary studies due to difficult comparability, they were excluded from the present systematic review (see [6] for results on these two primary studies). This resulted in 19 primary studies that were included in our first as well as this systematic review (Fig. 1).

### Study characteristics

Table 1 provides an overview of the study characteristics of the included articles. Table 2 highlights the included articles and their research results in more detail. Most studies were conducted in Germany, followed by Italy and Spain. Eleven studies dealt exclusively with migrants, seven with migrants and natives, and one study additionally examined refugees.

**Table 1:** Study characteristics of the included primary studies

	Number of primary studies (*n* = 19)
**Study design**	
Cross-sectional	15
Cohort	4
**Publication year**	
2016	3
2017	1
2018	5
2019	3
2020	1
2021	4
2022	2
**Study host country**	
Germany	5
Italy	4
Spain	3
Sweden	2
United Kingdom	2
Denmark	1
Finland	1
France	1
**Participants**	
Migrants	11
Migrants and natives	7
Migrants, refugees and natives	1
Occupations*	
Manufacturing industry (including construction)	4
Services	9
Agriculture, forestry, fishery	1
Mixed and/or unknown	8
**Outcome types**	
Primary	16
Secondary	1
Primary and secondary	2
**Outcome clusters**	
Psychological and psychiatric diagnoses (including suicide and suicide attempts)	1
Psychological and psychiatric symptoms	16
General distress	1
More general related constructs of mental health	3
**Working conditions**	
Work domain	5
Education-occupation match	4
Employment contract	9
Working schedule	9
Shift work	2
Rewards	6
Work ressources	5
Work strain/stress	4
Leadership style	2
Discrimination	7

*Categorization based on the microcensus model [34]

**Table 2:** Characteristics and research results of the included primary studies

Authors, publication year (study country)	Study design (representativeness)	Total sample (size, medium age (SD^a^), % women)	Migrant group(s) (size, percentage of the total sample, medium age (SD), % women)	Country (/-ies) of origin (percentage of all migrants)	Control group(s) (size, kind of control group(s), percentage of the total sample, medium age (SD), percentage of women)	Occupational group(s) (percentage of the total sample)	Work-related conditions (measurement instrument(s))	Mental health outcome(s) (measurement instrument(s))		Main results
								Primary	Secon-dary	
Braun et al., 2021 (Germany)	Cross-sectional study (not representative)	68 (10.3% women)	68 migrants (100%)	- Arab countries (57.4%) - Non-Arab countries (42.6%)	-	Urologists	- Contract type - Working schedule - Specialization - Work setting - Job position	Burnout (MBI^b^)	-	Having a permanent employment contract, working in managerial positions and working full-time as protective factors against the burnout dimension „Reduction of personal accomplishment“
Brendler-Lindqvist et al., 2022 (Sweden)	Cohort study (representative)	120 303 (50.2% women)	− 47 637 refugees (39.6%, 41.7% women) − 72 666 migrants (60.4%, 55.9% women)	- Eastern Europe, Russia and the post-Soviet republics (46.0%) - Western Europe, USA, Canada, Australia and New Zealand (11.5%) - Middle East (16.7%) - Horn of Africa and Sudan (3.9%) - South and Central America (4.5%) - East Asia (8.3%) - Other (9.0%)	-	-	Education-occupation-match (SSYK96^c^)	Hospitalization for psychiatric diagnoses (ICD-10^d^(	-	Over- and underqualification as risk factors for hospitalization for psychiatric diagnoses
Capasso, et al., 2016a (Italy)	Cross-sectional study (not representative)	250 (*M* = 43.2 (*SD* = 4.3), 100% women)	250 migrants (100%)	Eastern Europe (100%)	-	Eldercare (100%)	- Job type - Contract type - Working schedule - Work characteristics (JCQ^e^) - Effort-Reward-Imbalance (ERI^f^) - Work stress - Racial discrimination at work	- Interpersonal disorders (SCL-90-R^g^) - Anxious-depressive disorders (SCL-90-R^g^)	-	Positive association between high work demands and anxious-depressive as well as interpersonal disorders for collectivistic Eastern European migrants
Capasso et al., 2016b (Italy)	Cross-sectional study (not representative)	900	700 migrants (77.8%)	- Eastern Europe (35.7%, *M* = 43.2, 100% women) - Morocco (35.7%, *M* = 40.8, 10% women) - Ghana (28.6%, *M* = 38.8, 9% women)	200 natives (22.2%)	- Eldercare (27.8%) - Factory workers (38.9%) - Masons (33.3%)	- Job type - Contract type - Monthly income - Working schedule - Work characteristics (JCQ^e^) - Effort-Reward-Imbalance (ERI^f^)	- Interpersonal disorders (SCL-90-R^g^) - Anxious-depressive disorders (SCL-90-R^g^)	-	- Positive association between high work demands and anxious-depressive disorders for some natives - Negative association between high rewards and anxious-depressive disorders for collectivistic Maroccan migrants and natives - Positive associaton between high work demands and interpersonal disorders for collectivistic Ghanaian migrants and natives - Negative association between high rewards and interpersonal disorders for collectivistic Moroccan migrants and some natives - Negative association between high work resources and interpersonal disorders for some natives
Capasso et al., 2018a (Italy)	Cross-sectional study(not representative)	900 (*M* = 41.4 (*SD* = 4.1), 32.6% women)	700 migrants (77.8%)	- Eastern Europe (35.7%) - Morocco (35.7%) - Ghana (28.6%)	200 natives (22.2%)	- Eldercare (27.8%) - Factory workers (38.9%) - Masons (33.3%)	- Job type - Contract type - Working schedule - Work characteristics (JCQ^e^) - Effort-Reward-Imbalance (ERI^f^) - Work stress - Racial discrimination at work	- Interpersonal disorders (SCL-90-R^g^) - Anxious-depressive disorders (SCL-90-R^g^)	-	- Positive association between high work demands and interpersonal as well as anxious-depressive disorders for all workers - Negative association between high rewards and interpersonal disorders for all workers - Positive association between work stress and anxious-depressive disorders for all workers - Positive association between racial discrimination and interpersonal as well as anxious-depressive disorders for all workers - Higher risk for interpersonal disorders for collectivistic Moroccan and Ghanaian migrants - Higher risk for anxious-depressive disorders for collectivistic Eastern European migrants and some natives
Capasso et al., 2018b (Italy)	Cross-sectional study (not represen-tative)	250 (*M* = 40.8 (*SD* = 3.5), 10% women)	250 migrants (100%)	Morocco (100%)	-	Factory workers (100%)	- Job type - Contract type - Working schedule - Work characteristics (JCQ^e^) - Effort-Reward-Imbalance (ERI^f^) - Work stress - Racial discrimination at work - Income level	- Interpersonal disorders (SCL-90-R^g^) - Anxious-depressive disorders (SCL-90-R^g^)	-	- Positive association between high work demands and work stress for collectivistic Moroccan migrants - Positive association between racial discrimination and interpersonal disorders - Negative association of high rewards and interpersonal as well as anxious-depressive disorders - Positive association between high work demands in addition to racial discrimination and interpersonal disorders - Higher risk for interpersonal disorders
Espinoza-Castro et al., 2019 (Germany)	Cross-sectional study (not representative)	282 (39.4% women)	282 migrants (100%)	- Andean Community (55.7%) - Other South American Countries (26.2%) - Mexico and Central America (18.1%)	-	-	- Education-occupation-match (ISCO-08^h^) - Violence at the workplace (physical violence and sexual harassment)	Common mental disorders (GHQ-12^i^)	-	- Overqualification as risk factor for common mental disorders - Positive association between violence at the workplace and common mental disorders
Espinoza-Castro et al., 2021 (Germany)	Cohort study (not representative)	189 (89.4% women)	189 migrants (89.4% women)	- Colombia (49.2%) - Mexico and Central America (27.0%) - South America without Colombia (12.7%) - Spain (9.5)	-	Au pairs (100%)	- Working schedule - Working on holidays - Days off per week - Existence of a contract - Extra hours - Additional jobs - Physical violence by host children - Verbal offenses - Violence at the workplace (physical violence and sexual harassment)	Depressive symptoms (PHQ-9^j^)	-	- Working more than 40 h per week as predictor for depressive symptoms for collectivistic Latin American migrants - Suffering physical violence by host children as predictor for depressive symptoms
Gosselin et al., 2022 (France)	Cross-sectional study (representative)	19 211 (48.2% women)	898 migrants (4.7%)	- EU (31.7%) - Africa (43.3%) - Not EU, not Africa (24.9%)	18 313 natives (77.1%)	-	- Type of work domain - Contract type - Work sector - Worksite size - Night work - Job strain (JDCS model^k^) - Iso strain	Anxiety (GAD-Mini^l^)	-	- Positive association between job strain and anxiety disorder among native French and some migrant groups - Positive association between iso strain and anxiety among native French and some migrant groups
Holten et al., 2018 (Denmark)	Cohort study (not representative)	2 947	111 migrants (3.8%, *M* = 46.0, 92.0% women)	- Europe (70.8%) - North America (0.9%) - South and Central America (3.8%) - Africa (7.5%) - Asia (11.3%) - Middle East (5.7%)	2 836 natives (96.2%, *M* = 48.0, 98.0% women)	Eldercare (100%)	Transformational leadership (GTL^m^)	-	Well-being (WBI-5^n^)	Transformational leadership as a predictor for positive change in well-being for native Danes, but not for migrants
Hultin et al., 2016 (Sweden)	Cohort study (representative)	23 952 (56.1% women)	3 349 migrants (14.0%)	- Nordic (36.2%) - Europe (27.3%) - Non-Europe (36.5%)	20 603 natives (86.0%)	-	Education-occupation-match (SSYK96^c^)	Common mental disorders (GHQ-12^i^)	-	Over- as well as underqualification no risk factor for psychological distress, neither for natives nor for migrants
Martynowska et al., 2020 (UK)	Cross-sectional study (representative)	551 (*M* = 33.0 (*SD* = 7.7), 75.0% women)	551 migrants (100%)	Poland (100%)	-	-	- Financial situation - Perceived change in attitude or behavior of supervisors - Perceived change in attitude or behavior of co-workers	Perceived stress (PSS-Mind Garden^o^)	- Psycho-logical well-being (PWB^p^) - Life satisfaction (SWLS^q^)	- Positive association between negative change in attitude or behavior of supervisors or co-workers and perceived stress for individualistic Polish migrants - Negative association between perceived stress and psychological well-being and life satisfaction
May et al., 2021 (Germany)	Cross-sectional (not representative)	81 (8.6% women)	81 migrants (100%)	-	-	Urologists	- Contract type - Working schedule - Specialization - Work setting - Job position	Burnout (MBI^b^)	-	Being employed as senior or chief physician as protective factor against the burnout dimension „Reduction of personal accomplishment“
Nie & Lämsä, 2018 (Finland)	Cross-sectional (not representative)	117 (41.0% women)	117 migrants (100%)	China (100%)	-	Employees in knowledge-based organizations (100%)	- Paternalistic leadership of supervisor (PLS^r^)	Burnout (MBI^2^)	-	- Negative association between benevolence (as an aspect of the paternalistic leadership style) and burnout for collectivistic Chinese migrants - Positive association between authorianism (as an aspect of the paternalistic leadership style) and burnout
Ramos Villagrasa & García Izquierdo, 2018 (Spain)	Cross-sectional (not representative)	310	132 migrants (42.6%, *M* = 36.1 (*SD* = 10.0), 56.1% women)	Latin America (72.7%) - Non-communitarian Europe (14.4%) - Africa (6.1%) - Other cultures from all over the world (6.8%)	178 natives (57.4%, *M* = 27.0 (*SD* = 9.4), 57.4%, 58.5% women)	- Service sector - Industry sector - Con-struction sector - Agriculture and fishing (0.8%)	- Type of work domain - Safety climate (Attitudes to Safety Scale)	Common mental disorders (GHQ-12^i^)	-	- Positive correlation between the communication and individual responsibility dimension of safety climate and well-being for migrants and natives - Positive correlation between the goal dimension of safety climate and well-being for migrants - Positive correlation between the individual responsibility dimension of safety climate and well-being for natives - Dimensions of safety climate not as important predictors of well-being
Rhead et al., 2021 (UK)	Cross-sectional (not representative)	931 (76% women)	328 migrants (35.27%)	-	603 natives (64.8%)	Healthcare workers (100%)	- Occupational group - Personal experience of discrimination at work - Witnessing discrimination at work - Personal experience of bullying/ harassment at work - Witnessing bullying/ harassment at work	- Depressive symptoms (PHQ-9^j^) - Generalized anxiety (GAD-7^s^) - Somatisation symptoms (PHQ-15^t^)	-	- Positive association between personal experience of discrimination and bullying/harassment (but not witnessing) and probable anxiety or depression (even after adjusting for migration status) - Positive association between personal experience of bullying/harassment as well as witnessing discrimination and moderate or severe somatic symptoms (even after adjusting for migration status) - Positive association between personal experience of discrimination and somatic symptoms (but not after adjusting for migration status)
Ronda-Pérez et al., 2019 (Spain)	Cross-sectional (not representative)	130	102 migrants (78.5%, 59.8% women)	Colombia, Ecuador	28 natives (21.5%, 50.0% women)	-	- Occupational social class - Working schedule - Formality of employment - Shiftwork - Physical demands at the work place - Income level that precludes covering unforeseen expenses	Common mental disorders (GHQ-12^j^)	-	- Higher incidence of common mental disorders among natives than collectivistic Latin American migrants independently of the working schedule (≤ 40 h per week or > 40 h per week) - Higher incidence of common mental disorders among natives than migrants with better working conditions (formal employment, no shift work, no physical demands, enough salary to cover unforeseen expenses)
Virga & Iliescu, 2017 (Spain)	Cross-sectional (not representative)	477 (*M* = 32.0 (*SD* = 7.2), 29.0% women)	477 migrants (100%)	Romania (100%)	-	Blue-collar workers in construction work or agriculture (100%)	Job insecurity (JIS^u^)	- Burnout (MBI-General Survey^b^) - Mental health complaints (5-Item Scale)	-	Positive association between job insecurity and burnout as well as mental health complaints for collectivistic Romanian migrants
Wassermann & Hoppe, 2019 (Germany)	Cross-sectional (not representative)	176 (*M* = 35.3 (*SD* = 7.9), 53.4% women)	176 migrants (100%)	Italy (100%)	-	-	- Working schedule - Perceived overqualification (SPOQ^v^)	Depressive symptoms (CES-D^w^)	Life satisfaction (SWLS^q^)	- Positive association between perceived overqualification and depressive symptoms for individualistic Italian migrants - Negative association between perceived overqualification and life satisfaction

^a^Standard Deviation; ^b^Maslach-Burnout Inventory; ^c^Swedish Standard Classifications of Occupations (national adaptation to the International Standard Classification of Occupations (ISCO-88); ^d^International Classification of Diseases, version 10; ^e^Job Content Questionnaire; ^f^Efford-Reward-Imbalance-Scale; ^g^Symptom Checklist 90 R; ^h^International Standard Classification of Occupations; ^i^General Health Questionnaire-12; ^j^Patient Health Questionnaire-9; ^k^Karasek’s Job-Demand-Control-Support Model; ^l^Generalized Anxiety Disorder, Mini International Neuropsychiatric Interview; ^m^Global Transformational Leadership Scale; ^n^WHO-5-Well-being Index; ^o^Perceived Stress Scale– Mind Garden; ^p^Scale of Psychological Well-being; ^q^Satisfaction with Life Scale; ^r^Paternalistic Leadership Scale; ^s^Generalized Anxiety Disorder Scale-7; ^t^Patient Health Questionnaire-15; ^u^Qualitative job insecurity scale and quantitative Job Insecurity Scale; ^v^Scale of Perceived Overqualification; ^w^Short form of the Center for Epidemiological Studies Depression Scale

Different research questions in the same population were addressed in two studies [35, 36], subpopulations of those samples were examined in further two studies [37, 38]. The same participant pool was used in two other studies, which probably has lead to some overlap [39, 40].

### Quality appraisal

Tables 3 and 4 present the results of the study quality appraisal of cross-sectional and cohort studies. A minimum score of 3 and a maximum score of 8 was found. Among cross-sectional studies, most were of moderate quality (*n* = 12), some were of low quality (*n* = 3). Among cohort studies, one was of high quality, one of low quality and the rest of moderate quality (*n* = 2).

**Table 3:** Assessment of the methodological quality of cross-sectional studies

Authors, year	Selection			Comparablity	Outcome			Total score (out of 9)	Validity of outcome measurements
	Representa-tiveness of the sample Maximum: *	Sample size Maximum: *	Comparability between respondents and non-respondents Maximum: *	Control of confounders Maximum: **	Assessment of the outcome Maximum: **	Statistical test Maximum: *	Ascertainment of the outcome measurement Maximum: *		
Braun et al., 2021	-	-	-	**	-	*	*	****^a^	Validated in German^b^
Capasso et al., 2016a	-	-	-	**	-	*	*	****	Validated in Italian^b^
Capasso et al., 2016b	-	-	-	**	-	*	*	****	Validated in Italian^b^
Capasso et al., 2018a	-	-	-	**	-	*	*	****	Validated in Italian^b^
Capasso et al., 2018b	-	-	-	**	-	*	*	****	Validated in Italian^b^
Espinoza-Castro et al., 2019	-	-	-	**	-	*	*	****	Validated in Spanish^c^
Gosselin et al., 2022	*	-	*	**	-	*	*	******	Validated in French^b^
Martynowska et al., 2020	*	-	-	-	-	*	*	***	All 3 validated in original language^d^
May et al., 2021	-	-	-	**	-	*	*	****	Validated in German^b^
Nie et al., 2018	-	-	-	**	-	-	*	***	Validated in English^c^
Ramos Villagrasa et al., 2018	-	-	-	**	-	*	*	****	Validated in Spanish^c^
Rhead et al., 2021	-	-	-	**	-	*	*	****	All 3 validated in English^c^
Ronda-Pérez et al., 2019	-	-	-	**	-	*	*	****	Validated in Spanish^c^
Vîrgӑ et al., 2017	-	-	-	**	-	-	*	***	MBI validated in Romanian^b^, MHI-5 validated in English^d^
Wassermann et al., 2019	-	-	-	**	-	*	*	****	All 2 validated in Italian^c^

^a^Interpretation: 0–3 stars: low methodological quality, 4–6 stars: moderate methodological quality, 7–9: high methodological quality; ^b^Not known if validated version was used; ^c^Validated version was used; ^d^at least validated in the original language

**Table 4:** Assessment of the methodological quality of cohort studies

Authors, year	Selection				Compara-bility	Outcome			Total score (out of 9)	Validity of outcome measure-ments
	Representa-tiveness of the exposed cohort Maximum: *	Selection of non-exposed cohort Maximum: *	Ascertain-ment of exposure Maximum: *	Presence of outcome of interest at start of study Maximum: *	Compara-bility of cohorts Maximum: **	Assessment of outcome Maximum: *	Follow-up time Maximum: *	Adequacy of follow-up Maximum: *		
Brendler-Lindqvist et al., 2022	*	*	*	-	**	*	*	*	********^a^	International classification system
Espinoza-Castro et al., 2021	-	*	-	-	-	-	*	*	***	Validated in Spanish^b^
Holten et al., 2018	-	*	*	-	**	-	*	*	******	Validated in original language^b^
Hultin et al., 2016	*	*	-	-	**	-	*	*	******	Validated in Swedish^c^

^a^Interpretation: 0–3 stars: low methodological quality, 4–6 stars: moderate methodological quality, 7–9: high methodological quality; ^b^Validated version was used; ^c^Not known if validated version was used

### Measurement tools

In terms of mental health outcomes, different validated scales were used (Table 2). Tables 3 and 4 present an evaluation of their validity.

Concerning measurement tools for the assessment of working conditions, established and partially validated questionnaires were used in 13 studies. The questionnaires assessed organizational working conditions (e.g., work domain, education-occupation mismatch, employment contract) and social conditions at work (leadership style, discrimination). Post-migratory stressors at work were not examined in primary studies.

### Sample characteristics

One or more explicitly selected migrant group(s) from (a) particular country/-ies/regions of origin were examined by eleven primary studies [14, 35–38, 41–46]. Eight primary studies examined migrants without a specific focus on their origin [39, 40, 47–52]. One study took refugees into account [47]. Migrants and refugees from different origins were examined by seven studies. There, percentage distribution of the migrants’ and refugees’ countries/regions of origin were reported [39, 40, 47–51]. Further two studies did not present countries/regions of origin at all [44, 52]. Asylum seekers were not considered. Table 5 shows detailed sample characteristics.

**Table 5:** Sample characteristics^a^

	Total	Migrants	Natives
**Sample size ranges in primary studies**, ***n***	68–120 303	68–120 303	28 − 20 603
**Sample size**, ***n*(%)**	170 625 (100.0)	127 864 (74.9)	427 61 (25.1)
**Gender**, ***n*(%)**			
Men	80 455 (47.2)	61 057 (49.5)^b^	345 (10.6)^c^
Women	90 170 (52.9)	62 232 (50.5)^b^	2 897 (89.4)^c^
**Age range in years** ^d^	15–68	17–68	18–65
**Mean age**	41.42^e^	36.46^f^	46.45^g^
**Migrants‘ countries of origin**^h^, ***n*(%)**			
Eastern Europe/Poland/Romania	1 278 (53.1)		
Latin America	384 (16.0)		
Morocco	250 (10.4)		
Ghana	200 (8.3)		
Italy	176 (7.3)		
China	117 (4.9)		

^a^ Those 1 576 participants who were examined several times were considered only once for the calculation of all characteristics; ^b^For 4 575 migrants, the gender distribution could not be calculated; ^c^For 39 519 natives, the gender distribution could not be calculated; ^d^The age range for 2 771 participants was not reported; ^e^The weighted total mean age could not be calculated for 165 264 participants; ^f^The weighted mean age of migrants could not be calculated for 125 717 participants; ^g^The weighted mean age of natives could not be calculated for 39 547 participants; ^h^Nine primary studies (*n* = 125 459 migrants) did not focus on explicit regions/countries of origin of migrants

Among the studies that examined specific migrant populations, both collectivistic (Individualism Score > 50) and individualistic (Individualism Score > 50) migrants can be found [53]. Most studies (*n* = 8) dealt with migrants from collectivistic countries [14, 35–38, 41, 44, 45], two with migrants from individualistic countries [43, 46] (Table 6).

**Table 6:** Migrants’ countries of origin classified according to Hofstede’s cultural categorization^a^

Collectivism		Individualism	
Country	Individualism Score^b^	Country	Individualism Score
China	20	Italy	76
Eastern Europe	43^c^	Poland	60
Morocco	46		
Ghana	15		
Ecuador	8		
Colombia	13		
Romania	30		
Latin America	21^d^		

^a^Ten primary studies contained information about countries of origin; ^b^The Individualism score ranges from 0 to 100; 0–49: collectivistic tendencies, 50–100: individualistic tendencies [53]; ^c^The arithmetic mean of all Eastern European countries was calculated: Belarus (collectivistic: 25), Bulgaria (collectivistic: 30), The Czech Republic (individualistic: 58), Hungary (individualistic: 80), Poland (individualistic: 60), Republic of Moldova (collectivistic: 27), Romania (collectivistic: 30), Russian Federation (collectivistic: 39), Slovakia (in the middle of both dimensions: 52), Ukraine (collectivistic: 25) [54]. ^d^The arithmetic mean of all Latin American countries was calculated: Argentina (collectivistic: 46), Bolivia (collectivistic: 10), Brazil (collectivistic: 38), Chile (collectivistic: 23), Colombia (collectivistic: 13), Costa Rica (collectivistic: 15), The Republic of Cuba (collectivistic, score unknown, estimated: 21), Dominican Republic (collectivistic: 30), Ecuador (collectivistic: 8), El Salvador (collectivistic: 19), Guatemala (collectivistic: 6), Honduras (collectivistic: 20), Mexico (collectivistic: 30), Nicaragua (collectivistic, score unknown, estimated: 21), Panama (collectivistic: 11), Paraguay (collectivistic: 12), Peru (collectivistic: 16), Puerto Rico (collectivistic: 27), Uruguay (collectivistic: 36), Venezuela (collectivistic: 12) [55]

### Comparisons between voluntary migrants and refugees

#### Sample characteristics

Only one longitudinal study compared migrants (family reunification migrants, labor migrants, others) and refugees in terms of their working conditions and mental health. The samples consisted of 47 637 refugees (40%) and 72 666 migrants (60%). Among them, 58% and 56% were women and 79% and 77% were between 30 and 49 years old. Refugees mainly originated from Eastern Europe, Russia and the post-Soviet republics (61%), followed by the Middle East (25%), whereas migrants mainly came from Eastern Europe, Russia and the post-Soviet republics (36%) and the West (19%) [47].

### Description of work-related conditions and their association with mental health

Only comparisons between voluntary migrants and refugees on one organizational condition could be made.

### Organizational conditions

#### Education-occupation mismatch

While 16% of refugees were engaged in work that was below or above their skill level, this was the case for 20% of family reunification migrants, for 23% of labor migrants, and 20% of other migrants. Among refugees, men were more likely to be overqualified for their recent occupation. Over- and underqualifiaction acted as risk factors for hospitalization for psychiatric diseases but migration status (refugee vs. family migrant vs. labor migrant vs. other) did not act as a moderator [47].

### Comparisons between migrants from collectivistic and migrants from individualistic countries of origin

#### Sample characteristics

Ten studies examined migrants from specific countries of origin (2 405 migrants, 1% of all migrants). Among them were eight studies that examined collectivistic migrants (*n* = 1 678, 70%) from China, Morocco, Eastern Europe, Ghana, Ecuador/Colombia, Romania and Latin America and two studies that examined individualistic migrants (*n* = 727, 30%) from Italy and Poland (Table 6). Among collectivistic migrants, 39% were female, among individualistic 70%. The weighted average age could be calculated only for a part of the collectivistic migrants (*n* = 1 177) and was 37.4 years. Among individualistic migrants, it was 33.6.

### Description of work-related conditions and their association with mental health

#### Organizational conditions

Comparisons between collectivistic and individualistic migrants were restricted to three organizational and one social condition.

##### Education-occupation mismatch

Two cross-sectional studies stated no differences on the association between overqualification and mental health. For both collectivistic Latin American and individualistic Italian migrants, overqualification showed an association with worse mental health (common mental disorders [41], higher depressive symptoms and lower life satisfaction [46]).

##### Working schedule

Five cross-sectional studies did not show any differences in working schedule between collectivistic and individualistic migrants. Collectivistic Eastern European, Moroccan and Ecuadorian/Colombian (though not Ghanaian) as well as individualistic Italian migrants mainly worked full-time [14, 35, 37, 38, 46]. Due to lack of data no association with mental health could be examined.

##### Rewards

Six cross-sectional studies examined rewards at work (intrinsic/extrinsic rewards referring to self-assessed esteem reward and job security prospects reward (Capasso et al. 2016a; Capasso et al. 2016b; Capasso et al. 2018a; Capasso et al. 2018b)). Collectivistic Ecuadorian/Colombian migrants were disadvantaged compared to individualistic Polish migrants. This is reflected in the fact that a relatively high proportion of collectivistic Ecuadorian/Colombian migrants reported not being able to cover unforeseen expenses [14], while a relatively low proportion of individualistic Polish migrants reported being in a poor financial situation [43]. Overall, rewards only showed a lower risk for anxious-depressive (depression, somatization, anxiety) and interpersonal disorders (insecurity in social contact, paranoid thoughts, compulsion, hostility) for collectivistic Moroccan migrants [35, 38], but not for collectivistic Eastern European [35, 37] and Ghanaian migrants [35]. However, not fulfilled rewards such as job insecurity or a poor financial situation showed an association with worse mental health for collectivistic Romanian (higher burnout rates and mental health complaints) [45] and individualistic Polish migrants (perceived stress) [43].

### Social conditions

#### Discrimination

Four cross-sectional studies reported prevalences of discrimination at work. While 26% of collectivistic Moroccan migrants reported racial discrimination [38] and 14% of the collectivistic Latin American migrants were affected by workplace violence [41], 9% and 14% of individualistic Polish migrants in Great Britain reported negative changes in the attitudes of colleagues and supervisors toward them since the BREXIT vote [43]. For most of the migrants discrimination experiences were associated with mental health problems (higher risk for interpersonal and anxious-depressive disorders [36, 38], common mental disorders [41], perceived stress, lower psychological well-being and life satisfaction [43]).

### Comparisons between host countries

#### Sample characteristics

Five studies were conducted in Germany, four in Italy, three in Spain, two each in the United Kingdom (UK) and Sweden, and one each in Denmark, Finland and France (Table 1). Most migrants were examined in Sweden (*n* = 123 652), followed by France (*n* = 898), the UK (*n* = 879), Germany (*n* = 796), Spain (*n* = 711), and Italy (*n* = 700). In Finland, *n* = 117 and in Denmark, *n* = 111 migrants were studied. The largest number of natives was considered in Sweden (*n* = 20 603), followed by France (*n* = 18 313) and Denmark (*n* = 2 836). In the UK, *n* = 603 natives were included in the comparisons, in Spain *n* = 206, in Italy *n* = 200, and in Germany none.

Description of work-related conditions and their association with mental health.

Comparisons between host countries were conducted for eight organizational and two social conditions.

#### Organizational conditions

##### Work domain

Five cross-sectional studies examined work domains. In Germany and the UK the majority of migrant workers held manual jobs [41] or were more likely to work in jobs associated with lower socioeconomic status [52]. The situation in Spain was described as inconsistent. One study described migrants and natives as equally represented in work domains [51], while another study showed that migrant workers were more likely to hold manual occupations than natives. Regardless of work domain natives were more affected by common mental disorders than migrants [14]. In France a mixed sample of migrants and natives tended to be employed more often as professionals and skilled workers than as unskilled workers. Lower job positions were associated with work strain and iso strain (when individuals are exposed to work strain but experience low social support at work). Most migrants worked in the private sector which was related to work strain, but not iso strain [48].

##### Education-occupation match

Two cross-sectional and two cohort studies stated that a non-negligible proportion of migrant and native workers in Sweden and Germany was affected by an education-occupation mismatch [41, 46, 47, 50]. Here, migrants were at risk [50]. Education-occupation mismatch was associated with mental health problems in most countries (risk of hospitalization for psychiatric diseases [47], common mental disorders [41, 50], depressive symptoms and lower life satisfaction [46]).

##### Employment contract

Eight cross-sectional and one cohort study explored employment contracts. The majority of a mixed sample of migrants and natives in Italy held temporary employment contracts [36]. However, migrants mainly held fixed-term contracts [35, 37, 38], while there was no consistent picture for natives [35]. In France a mixed sample of migrants and natives was mainly employed under permanent contracts. Contract type did not relate with either work strain or iso strain [48]. Migrants in Germany were also mainly employed under permanent contracts [39, 40] which was associated with reduced burnout-rates [39]. In another study almost half of migrant workers in Germany did not have a formal contract. The existence of a formal contract did not act as a risk factor for depressive symptoms [42]. The rate of informality was smaller in Spain. Here, no differences between migrant and native workers were found. Natives with formal employment were more affected by common mental disorders than migrants with formal employment [14].

##### Working schedule

Nine cross-sectional studies as well as one cohort study examined working schedule. Among migrants in Germany [39, 40, 46], among both migrants and natives in Italy [35–38] and Spain [14], the majority worked full-time. Full-time work was related to lower burnout-rates for migrant workers in Germany [39]. In Germany, almost one fifth of migrants worked more than 40 h per week [41, 42], not differing from natives [41]. Working more than 40 h per week was a risk factor for depressive symptoms for migrants [42]. In Spain the rate of migrants and natives working more than 40 h was higher, whereby migrants and natives did not differ. Regardless of weekly working hours, natives suffered more often from common mental disorders than migrants [14]. Most of migrants in Germany worked extra hours which did not play any predictive role for depressive symptoms [42].

##### Shift work

Two cross-sectional studies investigated shift work. The majority of migrants and natives in France were not engaged in night work. Night work was associated with work strain and iso strain [48]. Almost half of the migrants in Spain worked shifts (more often than natives). Natives and migrants who worked shifts did not differ in the prevalence of common mental disorders, while natives without shift work suffered more often from common mental disorders than migrants without shift work [14].

##### Rewards

Seven cross-sectional studies analysed rewards. In Italy migrants were severely disadvantaged in terms of financial compensation compared to natives [35]. Overall, for a mixed sample of natives and migrants, as well as for some of the individual migrant and native groups, a relationship of rewards with lower risk for interpersonal and/or anxious-depressive disorder was found [35–38]. In the UK less than one fifth of migrants reported a difficult financial situation which was associated with perceived distress [43]. In Spain more than one third of migrants was not able to handle unforeseen expenses which was more often the case for migrants than for natives. Natives with enough salary were more likely to suffer from common mental disorders than migrants with enough salary [14]. Furthermore, job insecurity was identified to be associated with burnout and mental health complaints among migrants [45].

##### Work resources

Five cross-sectional studies explored work resources which contain social support at work and job control [35–38]. In Italy a mixed sample of migrant and native workers with high work resources did not show lower risk of interpersonal or anxious-depressive disorders [36]. For none of the migrant groups [35, 37, 38], but for one native group did high work resources show an association with lower rates of interpersonal disorders [35]. In France, around one quarter of migrants as well as natives suffered from iso strain (as a non-fulfilled resource) which was associated with anxiety disorders among natives, but not all migrant groups [48].

##### Work strain/stress

Four cross-sectional studies examined work strain or work stress. In France, one third of natives and one fifth to almost one half of migrants suffered from work strain. Work strain was associated with anxiety disorders for some native and some migrant groups [48]. In Italy, perceived work stress was associated with anxious-depressive disorders for a mixed sample of migrants and natives, but not for the specific migrant groups [36–38].

#### Social conditions

##### Leadership style

One cohort and one cross-sectional study analysed leadership styles. In Denmark, the transformational leadership style was studied. It describes leaders who strive to satisfy the higher order needs of their subordinates. Correspondingly, this leadership style implies that leaders as well as employees motivate each other [56]. A transformational leadership style was an important factor in maintaining well-being for natives, but not for migrants [49]. In Finland, the paternalistic leadership style was taken into account. It consists of three components: benevolence, morality and authoritarianism. While belevolence is characterized by leaders’ behaviors that relate to an individual and comprehensive care for the work and welfare of subordinates, morality describes leaders’ behavior that stands for the moral virtues of the supervisor. Authoritarianism includes the authority of the supervisor over employees, which demands their full respect and deference [57]. Authoritarianism was found to have a positive and benevolence was found to have a negative association with burnout [44].

##### Discrimination

Six cross-sectional and one cohort study examined discrimination experiences at work. Among migrants in Italy, 26% had been affected by racial discrimination [38]. For most migrant and native workers those discrimination experiences were associated with either interpersonal or anxious-depressive disorders or both [36–38]. Among migrants in Germany, 6 to 14% had been affected by workplace violence [41, 42]. Further 30% and 23% of migrant au-pairs suffered from physical violence by host children and verbal offenses at the workplace [42]. Those experiences were associated with common mental disorders and depressive symptoms for migrants [41, 42]. Physical violence by host children acted as predictive risk factor for depressive symptoms [42]. Among natives and migrants in the UK, 21% reported personal discrimination experiences and 44% reported personal bullying/harassment experiences. Migrants were more likely to personally experience discrimination and bullying/harassment and to witness colleagues being victims of discrimination. Personal experiences and partly witnessing experiences were associated with probable anxiety or depression and moderate to severe somatic symptoms for a mixed sample of migrants and natives [52]. However, only 9% of the migrants in the UK reported negative changes in the attitudes of colleagues and 14% negative changes in the attitudes of supervisors toward them since the BREXIT vote. Negative changes were related to perceived distress, and lower psychological well-being and life satisfaction [43].

## Discussion

This systematic review stated a higher exposure of migrants than refugees to an education-occupation mismatch, a higher prevalence of unfavourable working conditions in migrants from collectivistic countries and differences on organizational conditions between host countries. An education-occupation mismatch, unmet rewards, discrimination, (in parts) a non-desirable contract type and (in parts) work strain were associated with poorer mental health.

### Comparisons between migrants and refugees

In terms of organizational conditions, voluntary migrants are more affected by education-occupation mismatch than refugees which tend to be more likely to have an occupation that matches their skill level. This could be due to the fact that only slightly less than one-third of the voluntary migrants had been living in the host country for 11 to 15 years, while this was true for two-thirds of the refugees [47]. Accordingly, it could be assumed that the refugees had either completed training in the host country within this time and correspondingly had better chances of finding a suitable occupation in the host country, as there were less problems with the recognition of foreign training degrees. Another possibility would be that they had initially worked in an occupation that did not correspond to their skill level, as their training abroad was not recognized, but were then able to move up in their occupation over time by gaining experience in working life in the host country and thus got a job that corresponded to their skill level. When interpreting these results, however, it should not be forgotten that only one primary study dealt with this aspect, so that the results must be interpreted very cautiously and the representativeness must at least be critically questioned.

Overall, education-occupation mismatch (especially overqualification) negatively influences mental health for all migrant groups.

### Comparisons between collectivistic and individualistic migrants

The comparisons on organizational conditions revealed that in terms of rewards, migrants from collectivistic countries are disadvantaged. It should be noted, however, that all studies were conducted in individualistic countries (Finland, Germany, Italy, UK) leading to a cultural mismatch for collectivistic migrants [53]. Since individualism is more common in developed countries and collectivism is more common in less developed countries [58], an education in a collectivistic country, due to its potentially lower quality, could on the one hand lead to a lack of vocational skills to perform a job with higher demands and higher reward levels in an individualistic country. An education in an individualistic country, however, might have imparted these skills, which is why individualistic migrants might have better chances of getting jobs with higher reward levels. On the other hand, training in a less developed country could lead to less recognition of that training in the more developed host country because of the potential lower quality already mentioned. This could force collectivistic migrants to take up work associated with lower reward levels even more often than individualistic migrants. Moreover, the success of integration (including in working life) may also be related to cultural origin. Following explanatory approaches of other research groups [59, 60] migrants from individualistic countries whose culture of origin more closely resembles that of individualistic countries might experience less acculturation stress and find it easier to integrate. This may improve work performance and thus the chance of higher reward levels. The different work-life needs that individualistic and collectivistic workers have and which are met to different degrees in countries of different cultural backgrounds, could also play a role in work performance. Since collectivistic migrants are more likely to perform better when they work in groups and their work is anonymous [12], they might perform worse in an individualistic society where it is expected that the best performance is achieved when working alone and one’s work is traceable. This could reduce their chance of getting an employment with high reward levels. In order to address the question of whether the similarity of cultural background between migrants and the host country is associated the quality of working conditions, it would be interesting in future studies to compare migrants migrating between Schengen member states [61] and migrants migrating from outside the Schengen area since Schengen member states should be similar especially in political terms. No differences were found with regard to working schedule. Thus, collectivistic as well as individualistic migrants work mainly full-time, equally often more than 40 h per week and are affected by overqualification.

Regarding social conditions at work, collectivistic migrants seem to be more frequently affected by discrimination. However, the fact that the primary studies examined different degrees of severity of discrimination experiences should not be ignored. While studies with collectivistic migrants dealt with racial discrimination and even workplace violence (physical, verbal or sexual), the primary studies with individualistic migrants only engaged in negative attitudes toward them by colleagues or supervisors after a political change. These differences in the severity of discrimination experiences make direct comparisons difficult. However, the fact that migrants from collectivistic countries report higher prevalences of more severe experiences of discrimination demonstrates that this migrant group represents a socially disadvantaged population. However, as mentioned above, it should be noted that primary studies on individualistic as well as those on collectivistic migrants were conducted in individualistic host countries. The more pronounced discrimination against collectivistic migrants could therefore again be due to a cultural mismatch, which is based on different needs for life in a society between individualistic and collectivistic persons [11]. These different and sometimes unmet needs could lead to conflict and result in experiences of discrimination.

In terms of the association of working conditions with mental health, it was found that for both collectivistic and individualistic migrants, unmet rewards, education-occupation mismatch, and discrimination were associated with poorer mental health.

## Comparisons between host countries

Some organizational conditions for migrants at work differ between host countries. For migrants in France, the possibility to be employed in higher-skilled work is higher than in Germany, UK and Spain. Migrants and natives in Spain and France face similar work position possibilities. In France, migrants and natives have better opportunities to receive favourable employment contracts than in Italy. In France and Spain, migrants and natives have the same opportunity to receive desirable employment contracts. In Italy and Spain, migrants have a similarly high chance of holding full-time jobs. In Germany and Spain, migrants and natives work more than 40 h per week to the same extent. In general, shift work is more common in Spain than in France, whereby migrants are more affected by shift work in Spain. Migrants in Italy and Spain are disadvantaged in terms of rewards, especially in terms of financial compensation, which is not the case for migrants in the UK.

In terms of social conditions, no differences were found between the host countries regarding discrimination. In Italy, the UK and Germany, migrants (and partially natives) experience negative attitudes toward them as well as discrimination and bullying/harassment, up to physical, verbal and sexual violence in the workplace. Differences in perceptions of leadership styles were difficult to identify, as the two studies from Denmark and Finland examined different leadership styles. On the whole, there are only marginal differences between the European host countries in terms of migrants’ experience of worse working conditions compared to natives. France stands out slightly from the other European host countries because here, migrants and natives more often have similar opportunities in the labor market and, according to the primary studies, worse working conditions are less common. In Spain, Italy and Germany, however, unfavorable working conditions were cited slightly more frequently. This could be due to the fact that France has a much longer tradition as a country of immigration [62, 63], and thus better functioning administrative procedures for integrating migrants into the labor market may already have been established there.

Regarding mental health, an education-occupation mismatch and (in parts) a non-desirable contract type are associated with worse well-being in most host countries, while rewards and work resources are (partly) related to better mental health, regardless of the host country. Further, work strain/stress is partly associated with worse well-being, independently from the host country. While a transformational leadership style of the supervisor, characterized by mutual motivation between employees and supervisors [56], plays an important role in maintaining mental health among natives but not among migrants [49], certain aspects of the paternalistic leadership style are associated with poorer mental health. The reason for this may be that a transformational leadership style is more likely to meet the needs of the individualistic native Danes [53] examined in this primary study [49], while it does not address the needs of the approximately 30% collectivistic heterogeneous migrant group [53]. The paternalistic leadership style, on the other hand, characterized by authority on the part of the supervisor and by control over the employees [57], shows, in part, a positive relationship with mental health for collectivistic migrants [44]. Thus, when interpreting these results, it is important to keep in mind that different leadership styles, due to their typical characteristics, meet different needs of individuals from different cultural origins and thus have different effects on mental health. Discrimination negatively relates to well-being, regardless of the host country.

Various reviews about working conditions of migrants that did not focus exclusively on European countries revealed similar results (Arici et al. 2019; Hargreaves et al. 2019; Sterud et al. 2018). Accordingly, Europe does not differ from other countries in terms of migration laws and migrants’ and refugees’ integration into the labor market. However, many reviews frequently report occupational accidents and physical injuries [5, 64, 65], which was not addressed in our review due to a lack of primary studies on this aspect.

### Strengths and limitations

An important strength of the present systematic review can be seen in the very strict and narrow inclusion and exclusion criteria. This particularly concerns the definition of “migrant”. No uniform definition of “migrant” is used internationally. In Germany, for example, “persons with a migration background” are understood to be people of the first and second migration generation [66]. In other countries, however, “migrant” is understood as only people of the first migration generation, while people of the second migration generation are defined as natives. These different definitions would have led to an impossible comparison between studies, as the definitions would have been mutually exclusive. Therefore, we introduced a single definition for “migrant” as first-generation migrants only. This resulted in the fact that studies that counted both first- and second-generation migrants as “migrants” and whose results between those groups could not be separated had to be excluded. Furthermore, studies that did not report a clear definition of “migrant” and for which even after contacting the authors did not yield the necessary information about the exact definition had to be excluded. Another strength can be seen in the fact that studies in which not all subjects were working at the time of the examination were excluded. This guaranteed that only participants who could make valid and realistic statements about their working conditions were included in the comparisons. However, cohort studies were also included, some of which contained subjects who were working at the first measurement point, but then left their employment during follow-up. In this case, it seemed more important to us to include valuable and relatively rare cohort studies that allow causal relationships, especially since it can also be assumed that a person who was working at the first measurement point would still be able to make valid and realistic statements about working conditions at the follow-up. Another strength can be seen in our broad language expertise. We were able to include primary studies in seven European languages, which should cover the vast majority of primary studies from Europe. Furthermore, the updated literature search guarantees that very recent studies on this topic were included.

However, limitations in the present systematic review have to be mentioned as well. The first limitation can be seen in the moderate methodological quality of a majority of the primary studies. The main weaknesses of the primary studies were identified as lack of representativeness of the sample, lack of justification of the sample size, insufficient comparability between respondents and non-respondents, and the assessment of only self-reported outcomes. This demonstrates that research of high methodological quality of migrants and their working conditions is missing until today, which should be considered in future research. A second limitation is the nature of the examined sample, which is referred to as a „hard-to-reach population“. This is because national registries about migrants and refugees/asylum seekers are lacking and that it is difficult to recruit this sample due to their lower willingness to participate in research studies [67]. This led to the fact that despite an extensive systematic literature search, only one primary study that examined refugees as a separate migrant group in the work setting was identified. The fact that migrants and refugees are a population group that is considered difficult to recruit ultimately leads to a fragmented picture of migrants and refugees in the European labor market [67]. Moreover, it should not be disregarded that the willingness to participate in scientific surveys might be lower among less integrated migrants/refugees, for example, out of mistrust and fear of stigma and privacy [68]. As a result, the primary studies examined mainly migrants/refugees who were already socially and economically well integrated. This compromises the representativeness of the findings. Accordingly, it must be assumed that mental burden (and possibly also working conditions) among migrants/refugees might be worse than could be shown in our systematic review. A third aspect to be criticized is the fact that very heterogeneous outcome measurement instruments were used in the primary studies. Mental health outcomes such as depression or well-being were assessed with different instruments, which complicates comparability between primary studies. Nevertheless, since only studies with measurement instruments that were validated at least in the original language were included, only reliable and valid measurement instruments were used. This should allow reliable conclusions. However, when using self-report measures, bias susceptibility should not be disregarded, as participants may exhibit specific response tendencies, for example, due to social desirability [69]. There was no obligation for validation for measurement instruments that assessed working conditions, which difficults comparibility between primary studies. Since our goal was to describe working conditions as broadly as possible, however, a large heterogeneity of measurement instruments can be seen as an advantage.

In terms of the cultural classification of migrants, other methods could also have been used, such as Inglehart’s and Wetzel’s cultural map [70], Schwartz’ values [71] or the recently published F-index by Mutukrishna and colleagues, which reflects the cultural distance between countries [72]. In this systematic review, however, the Hofstede classification [11] was chosen because it made sense to use a categorization system that was based on a similar population to the sample in this systematic review. Since the present study only included people who were currently working at the time the primary studies were conducted, and the sample that served as the basis for Hofstede’s theory only included people who were employed in various IBM subsidiaries in over 40 different countries [73], the categorization according to Hofstede was adequate due to the matching inclusion criterion that the subjects had to be currently working. However, other categorization suggestions could also be used in future studies. Using different indices to group cultures can provide information about different aspects of cultural imprinting. For example, different countries may be very similar in one cultural aspect but very different in other aspects [72]. Taking different cultural aspects into account could provide in-depth information on which cultural aspects and differences result in which disadvantages in the work setting and to what extent this leads to inequalities in mental health. Furthermore, instead of focusing solely on Hofstede’s Individualism Score, the other cultural dimensions proposed by Hofstede, namely Power Distance, Uncertainty Avoidance, Long Term vs. Short Term Orientation and Indulgence vs. Restraint [11], could also be taken into consideration in future studies to provide a detailed characterization of participants from different cultural backgrounds.

## Conclusion

The present systematic review revealed a lack of studies on working conditions of migrants and especially refugees in Europe and their association with mental health. There is a great need for more research in this area in the future. Migrants and refugees suffer from unfavourable working conditions in all European host countries. Migrants from collectivistic societies seem to be at particular risk. There are no major differences between the European host countries, but France stands out slightly in a direct comparison, as migrants and natives more often have similar opportunities for decent working conditions there. The majority of unfavorable working conditions was related to worse mental health. Due to the high proportion of migrants in the workforce in European countries, migrants’ (mental) health has ceased to be a niche topic, but a public health issue that should also be addressed at a political level. Labor law in European countries should generally pay attention to working conditions and control them in order to maintan and/or enhance workers’ mental health. Special attention should be paid to vulnerable population groups such as collectivistic migrants. At political level, this could be realized through a wider recognition of training/graduate degrees from abroad. At the organizational level, this could be achieved through anti-discrimination and team-building programs as well as workplace health promotion offerings in which, for example, (multilingual) information about employees’ rights is passed on. Furthermore, employers must be sensitized to the cultural origin of their employees and to linguistic and cultural barriers in order to be able to reduce these in a targeted manner.

### Electronic supplementary material

Below is the link to the electronic supplementary material.

Supplementary Material 1

## Data Availability

All data generated or analysed during this study are included in this published article and its supplementary files.
